# The Therapeutic Potential of Multipotent Mesenchymal Stromal Cell—Derived Extracellular Vesicles in Endometrial Regeneration

**DOI:** 10.3390/ijms24119431

**Published:** 2023-05-29

**Authors:** Gyuzyal Tabeeva, Denis Silachev, Polina Vishnyakova, Alexandra Asaturova, Timur Fatkhudinov, Antonina Smetnik, Madina Dumanovskaya

**Affiliations:** 1 National Medical Research Center for Obstetrics, Gynecology and Perinatology Named after Academician V.I. Kulakov of Ministry of Healthcare of Russian Federation, 117997 Moscow, Russia; p_vishnyakova@oparina4.ru (P.V.); a_asaturova@oparina4.ru (A.A.); a_smetnik@oparina4.ru (A.S.); m_dumanovskaya@oparina4.ru (M.D.); 2 A.N. Belozersky Institute of Physico-Chemical Biology, Lomonosov Moscow State University, 119991 Moscow, Russia; 3 Research Institute of Molecular and Cellular Medicine, Peoples’ Friendship University of Russia (RUDN University), 117198 Moscow, Russia; fatkhudinov-tkh@rudn.ru; 4 Avtsyn Research Institute of Human Morphology of Federal State Budgetary Scientific Institution Petrovsky National Research Centre of Surgery, 117418 Moscow, Russia

**Keywords:** regeneration, thin endometrium, Asherman’s syndrome, intrauterine adhesions, synechia, multipotent mesenchymal stromal cells, extracellular vesicles

## Abstract

Disruption of endometrial regeneration, fibrosis formation, and intrauterine adhesions underlie the development of “thin” endometrium and/or Asherman’s syndrome (AS) and are a common cause of infertility and a high risk for adverse obstetric outcomes. The methods used (surgical adhesiolysis, anti-adhesive agents, and hormonal therapy) do not allow restoration of the regenerative properties of the endometrium. The experience gained today with cell therapy using multipotent mesenchymal stromal cells (MMSCs) proves their high regenerative and proliferative properties in tissue damage. Their contribution to regenerative processes is still poorly understood. One of these mechanisms is based on the paracrine effects of MMSCs associated with the stimulation of cells of the microenvironment by secreting extracellular vesicles (EVs) into the extracellular space. EVs, whose source is MMSCs, are able to stimulate progenitor cells and stem cells in damaged tissues and exert cytoprotective, antiapoptotic, and angiogenic effects. This review described the regulatory mechanisms of endometrial regeneration, pathological conditions associated with a decrease in endometrial regeneration, and it presented the available data from studies on the effect of MMSCs and their EVs on endometrial repair processes, and the involvement of EVs in human reproductive processes at the level of implantation and embryogenesis.

## 1. Introduction

One of the most important conditions for a woman’s fertility is the functional potential of the endometrium, which ensures successful implantation of the embryo. This complex process requires synchronisation between a healthy embryo and a functioning endometrium. The endometrium is physiologically unique, because it is a tissue capable of restoring during each menstrual cycle without scar tissue formation [1]. The high frequency of intrauterine interventions contributes to damage to the basal layer of the endometrium, decreases angiogenesis, and causes neovascularization and inflammation [2]. The disruption of endometrial regeneration, the formation of fibrosis, and intrauterine adhesions associated with these changes lead to the development of “thin” endometrium and/or Asherman’s syndrome (AS) [3] and are a common cause of infertility and a high risk of adverse obstetric outcomes [4]. Currently, there are practically no effective pharmacological preparations with a high regenerative and anti-inflammatory potential [5]. At the same time, the approaches used (surgical adhesiolysis, anti-adhesive agents, and hormonal therapy) help to reduce the frequency of recurrence of intrauterine adhesions [5], but do not allow to solve the main problem—the restoration of the regenerative properties of the endometrium and its functional activity.

Today’s accumulated experience with cell therapy using multipotent mesenchymal stromal cells (MMSCs) demonstrates their high regenerative and proliferative properties in traumatic or ischemic tissue damage. A number of experimental and clinical studies demonstrated their effect on stimulating regenerative and angiogenesis processes to restore the functional potential of the endometrium [6,7,8,9,10]. Despite many years of research on the therapeutic potential of MMSCs, the mechanisms of intercellular communication involving these cells and their contribution to regenerative processes remain poorly understood. One of these mechanisms is based on the paracrine effects of MMSCs, which are associated with the stimulation of cells of the microenvironment by secreting extracellular vesicles (EVs) into the extracellular space. EVs are nanotransporters for biologically important molecules involved in the maintenance of physiological tissue homeostasis. EVs, whose source is MMSCs, are able to stimulate progenitor cells and stem cells in damaged tissues and exert cytoprotective, antiapoptotic, and angiogenic effects [4,11].

This review describes the regulatory mechanisms of endometrial regeneration, pathological conditions associated with a decrease in endometrial regeneration, and it presents the available data from experimental studies on the effect of MMSCs and their EVs on endometrial repair processes, and the involvement of EVs in human reproductive processes at the levels of implantation and embryonic development [12].

## 2. Endometrial Regeneration

Among the tissue components of the endometrium, a single-layered prismatic epithelium, loose fibrous connective tissue, and uterine glands are distinguished. At the cellular level, the endometrium consists of epithelial and stromal components, immune cells (T-lymphocytes, NK cells, granulocytes, macrophages, B-lymphocytes, mast cells, etc.) and blood vessels. The epithelium consists of luminal and glandular cells, and the stroma consists of fibroblast-like cells (mesenchymal supporting cells, fibroblasts), argyrophils, and collagen fibres. The endometrium consists of two layers: the basal layer and the functional layer. The functional layer of the endometrium contains luminal (cubic) and glandular (columnar) epithelial cells surrounded by a loose stroma traversed by spiral arterioles. It is sensitive to sex hormones, which is why its thickness and structure vary according to the phase of the menstrual cycle. Its thickness varies from 1 mm (in the early proliferative phase) to 8–14 mm (in the secretory phase). In the follicular phase, the luminal epithelium contains many ciliated cells, whose function is to remove cellular secretions, which participate in sperm kinetics and their implantation in the oocyte. The nonciliated (secretory) cells of the functional layer bear numerous, rather long microvilli, more of which can be observed during the proliferative phase of the cycle and during the implantation window [13]. The luminal epithelium plays a central role in determining endometrial receptivity [14]. Under the action of estradiol, the functional epithelium actively undergoes proliferation processes, while under the action of progesterone, differentiation processes occur. During menstruation, the functional layer is shed. The basal layer is structurally more stable throughout the menstrual cycle and is less sensitive to sex hormones [15]. It is adjacent to the myometrium and consists of cells of the glandular epithelium, dense stroma, and short straight arteries. Its thickness is 1–1.5 cm. The glands located at the base of the basal layer merge into the functional layer. The function of the basal layer of the endometrium is the cyclic formation of a new functional layer after menstruation [16].

Overall, women have up to 450 cycles throughout the reproductive period during which, under the influence of hormonal, pro-inflammatory and anti-inflammatory, epigenetic signals and with the participation of stem and progenitor cells, the functional layer of the endometrium is rebuilt after menstruation [17,18,19,20]. This remodelling involves cell proliferation, differentiation, detachment, and cell regeneration [21].

The earliest phase of endometrial repair begins within 36 h of the onset of menstruation and lasts for 48 h [22]. The drop in progesterone levels during the secretory phase of the cycle in the absence of pregnancy triggers a consistent sequence of interdependent pro-inflammatory events in the endometrium. In decidualized stromal, vascular, and epithelial cells, there is a decrease in prostaglandin metabolism, suppression of expression of the enzyme Su-peroxide dismutase, loss of protection from reactive oxygen species (ROS), leading to activation of nuclear factor-κB (NF-κB) expression, transcription of target genes, and an increase in the synthesis of pro-inflammatory prostaglandins, cytokines, chemokines, and vasoconstriction of spiral arterioles. The hypoxia produced under these conditions promotes activation of NK cells and mast cells, recruitment of neutrophils and eosinophils, differentiation of monocytes into pro-inflammatory M1 macrophages, resulting in the release of pro-inflammatory cytokines, growth factors (IL-1ß, TNF-α and IL-6), matrix metalloproteinases (MMPs), which contribute to the clearance of tissue degradation products, tissue breakdown, necrosis of vessel walls, and bleeding characteristic of menstruation [21]. In parallel, immediately after the detachment of the functional layer of the endometrium, the following processes occur: Activation of neutrophils, transition of macrophages to phenotype 2 (M2—anti-inflammatory), secretion of anti-inflammatory cytokines and growth factors (IL -10, TGF-1 and VEGFB), all of which determine the initial phase of tissue recovery after menstruation [21,23].

Currently, several mechanisms of endometrial tissue repair were described: reepithelialization (proliferation and migration of the remaining glandular and luminal epithelial cells), cellular transdifferentiation of stromal cells into epithelial cells (mesenchymalmal-epithelial transition (MET)), and realization of the regenerative potential of the stem cell populations of the basal and functional layers of the endometrium with the possible participation of bone marrow-derived stem cells (BMDSCs) [19,24].

Studies showed that endometrial cells have the ability to differentiate into both stromal and epithelial cells, indicating the role of MET in the regenerative process. Experimental and clinical data show that the expression of genes (WT1, Snai 1, 2, 3, Cdn1, MMP3, TWIST, etc.) and cell adhesion molecules (cadherins, fibronectin, etc.) changes during endometrial regeneration toward a decrease in stromal markers and an increase in epithelial markers involved in MET [15]. These cellular processes promote cyclic repair of endometrial cells, which includes both epithelial cell migration and mesenchymal cell differentiation. The accumulated data suggest that the regulation of MET occurs with the participation of the coordinated work of several signaling pathways, such as TGFβ/SMAD, WNT, PI3K/AKT/mTOR, MARK/ERK, Jak- STAT, Hedgehog, Notch, Hippo, the exact mechanisms of which were not yet fully investigated [25].

Among endometrial stem cells, epithelial progenitor cells, mesenchymal stromal cells, and side population cells are distinguished.

**Figure 1:**
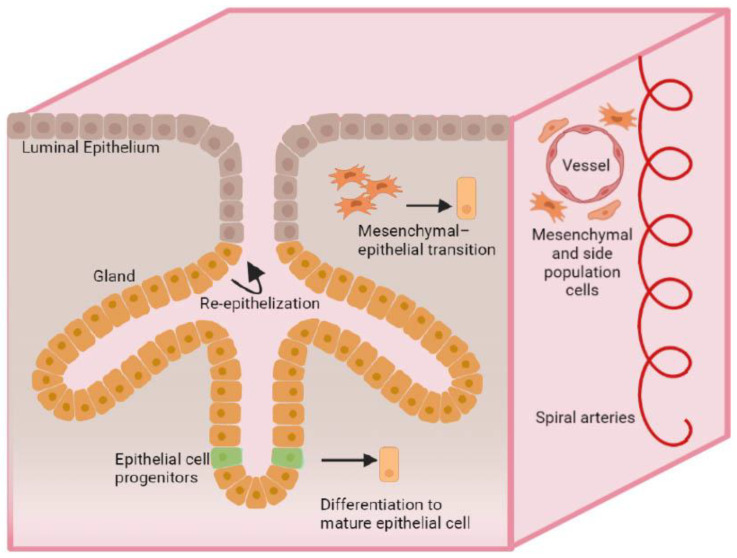
A schematic illustration of mechanisms of endometrial regeneration.

Epithelial progenitor cells are located in the luminal epithelium of the glands of the basal layer of the endometrium [18]. They are characterized by high telomerase activity and the absence or low expression of estrogen receptor α (ERα). However, they were observed to be able to proliferate in response to estradiol stimulation by interacting with ERα-expressing cells [18], which transmit signals through the production of growth factors (epithelial growth factor (EGF), transforming growth factor α (TGFα), and fibroblast growth factor 2 (FGF2)). The Wnt/β-catenin signaling pathway is also an important regulator of epithelial progenitor cell maintenance and differentiation [26]. According to various sources, the marker for epithelial progenitor cells is stage-specific embryonic anti-gene-1 (SSEA-1), N-cadherin, AXIN2 [18,27], expressing stem cell factor receptor c-Kit (CD117), OCT-4 [28]. Mesenchymal stem cells play an important role in the regeneration of the stromal component of the endometrium, as well as in immune regulation and angiogenesis, and are involved in placentation [29]. They are also associated with various signaling pathways such as Notch, TGFβ, and Hedgehog [30]. Surface markers of a small population of mesenchymal stem cells (CD146 + PDGFRβ+, SUSD2+) show their perivascular localization in the functional and basal layers of the endometrium and are also found in menstrual blood [31,32]. The cells of the lateral population express different types of cell markers, namely the undifferentiated c-KIT and OCT-4 markers, the endothelial cell markers CD31 and CD34, EMA and the mesenchymal stem cell markers CD90, CD105, and CD146 [33]. They are predominantly endothelial cells and, like the SUSD2+ mesenchymal stromal cells, express neither ERα nor progesterone receptors (PR), but estrogen receptors ß (ERβ). Human endometrial cells were differentiated in xenografts of the side population cells, which consisted mainly of stromal and vascular tissue, with fragments of epithelial glandular structures [34,35] (Figure 1).

The detection of BMDSCs in the endometrium suggests that they play the main function in regeneration. A number of researchers pointed to the ability of BMDSCs to transdifferentiate into epithelial, stromal, and endothelial cells of the endometrium [36,37]. Recently, however, there were data indicating that only bone marrow-derived immune cells are present in the endometrium, refuting the contribution of BMDSCs to endometrial regeneration [24,38].

Damage to the basal layer of the endometrium and the development of subsequent inflammation contribute to the suppression of regeneration, reduction in angiogenesis, neovascularization, and formation of fibrosis [2,39,40]. Confirmation of microcirculatory insufficiency is a general decrease in vascular density in the endometrium in such pathologies due to suppression of the expression of vascular (VEGF, bFGF) and other growth factors (TGF-β, PDGF, CCN2, etc.), leading to the development of hypoxia and tissue ischemia. At the same time, the endometrium loses its functional activity and the ability to respond to the action of sex steroids [41]. One of the main aspects of impaired endometrial regeneration is a change in the balance of MET, which contributes to the disruption of cell differentiation and activation of proliferation of fibroblasts and the extracellular matrix [42], leading to the loss of epithelial cells, replacement of the stroma by fibrous tissue, and the formation of synechiae in the uterine cavity [41]. Experimental and clinical data show that the expression of genes (WT1, Snai 1, 2, 3, Cdn1, MMP3, TWIST, etc.) and cell adhesion molecules (cadherins, vimentin, fibronectin, etc.) changes when endometrial regeneration fails, while stromal markers involved in MET increase and epithelial markers decrease [15]. In recent years, scientists became interested in studying the influence of the Hippo pathway on the regulation of TGFβ expression, a key mediator of fibrosis formation. A number of studies showed that activation of the Hippo pathway can lead to changes in mechanotransduction in fibroblasts, phosphorylation, and accumulation of the important transcriptional coactivators TAZ (transcriptional coactivator with PDZ-binding motif)/YAP (Yes-associated protein) in the cell cytoplasm, and a reduction in TGFβ-induced expression of profibrotic genes [43]. In addition, the duration of the immune response to injury and the transition from one macrophage phenotype to another may also determine the outcome of tissue healing and fibrosis formation. In the first phase of the adaptive M1 immune response, macrophages synthesize pro-inflammatory cytokines and growth factors (IL-1ss, TNF-α, and IL-6), contributing to the clearance of tissue degradation products. In the second phase, M2 macrophages secreting anti-inflammatory cytokines and growth factors (IL-10, TGF-1 and VEGFB) are involved in the regeneration of endometrial tissue [23]. Apparently, disruption and loss of stem and progenitor cells, aberrant regulation of signaling pathways, and maintenance of immune response activation toward accumulation of the M1 phenotype of macrophages in the endometrium lead to suppression of MET processes and loss of regenerative capacity and activation of fibrosis.

## 3. Pathology of the Endometrium Associated with Its Impaired Regeneration

### 3.1. “Thin” Endometrium

“Thin” endometrium refers to a condition in which the thickness of the endometrium is below the threshold for pregnancy. In clinical practice, ART (assisted reproductive techniques) programs with endometrial thickness (ET) less than 7 mm during the “window” of implantation have a reduced likelihood of pregnancy and are associated with early and late obstetric complications [44,45,46], explained by impaired receptivity and angiogenesis, inflammation, and a reduction in the efficacy of antioxidant mechanisms in the endometrium [47,48,49]. However, there is no consensus on the threshold of endometrial thickness at which pregnancy is impossible. In the studies of Check JH (2003) and Sundstroem P (1998), cases of pregnancy with the birth of healthy children were demonstrated in both natural and stimulated cycles with a ET of 4 mm [50,51]. However, since ET often determines the outcome of implantation and pregnancy, it is now recognized as an important surrogate marker of endometrial receptivity [52].

Causes of a “thin” endometrium include damage to the basal layer of the endometrium, hypoestrogenic conditions, prolonged use of oral contraceptives, stimulation of ovulation with clomiphene citrate, radiation therapy [53,54,55], impaired estrogen signaling due to estrogen receptor dysfunction (Erα- and β-polymorphisms) [56,57], while in some cases, the causes remain unknown.

Although there is no consensus on the threshold of ET below, which is defined as “thin,” the most commonly reported value in ultrasound examinations is 7 mm [44,46]. In ART programs, 2.4% were found to have an ET ≤ 7 mm, which is associated with a significantly lower likelihood of clinical pregnancy, live birth, and increased likelihood of miscarriage [44].

### 3.2. Asherman’s Syndrome

Asherman’s syndrome (AS) is a symptom complex characterized by partial or complete obliteration of the uterine cavity and/or cervical canal due to damage to the basal layer of the endometrium. Clinically, AS is manifested by hypo-menstrual syndrome, amenorrhea, infertility, and miscarriages. The most common cause of AS (up to 66%) is endometrial curettage, which is performed to remove the fetal oocyte during a non-developing pregnancy, the remnants of the fetal oocyte after spontaneous abortion, and placental tissue after delivery [2,58]. The technical characteristics of such intrauterine procedures (the impossibility of gentler methods of emptying the uterine contents), the high incidence of endometritis [58], and hypoestrogenism contribute to poor regeneration of the endometrium and the development of fibrous tissue. Damage to the basal layer of the endometrium may also occur after diagnostic curettage of the uterine cavity for abnormal uterine bleeding, after uterine surgical procedures (myomectomy, metroplasty, cervical conization), after suturing of the uterus to stop postpartum bleeding, and after uterine artery embolization [59]. The role of Mycobacterium tuberculosis in the development of genital tuberculosis with further AS with complete obliteration of the uterine cavity was also demonstrated [60].

Several classifications of intrauterine adhesions were proposed based on the prevalence of adhesions and different imaging modalities of the uterine cavity [61,62]. The most commonly used classifications are those of the American Fertility Society [63], the European Society for Gynaecologic Endoscopy [64], and a number of others. One of the most objective classifications is that of Nasr (2000) [65]. It is based not only on the presence of synechiae but also on the clinical features of AS (menstrual irregularities and reproductive history) [59]. Since there is no consensus on the optimal classification system for AS, it is difficult to perform meta-analyses and select the optimal treatment method.

For the initial diagnosis of AS, an echographic examination of the pelvic organs is performed. Other imaging modalities include sonohysterography (SHG), including 3D SHG, hysterosalpingography, and magnetic resonance imaging. According to the American Association of Gynecologic Laparoscopists (AAGL, 2010) practice guidelines for the management of women with intrauterine adhesions, hysteroscopy is the method of choice for the diagnosis of AS with a high level of evidence [66].

The treatment of AS includes surgical destruction of intrauterine adhesions in the first stage. This approach is more effective for mild to moderate intrauterine adhesions. The frequency of recurrence after resolution of intrauterine adhesions was 24%, and in severe forms of SA—63% [41]. Dissection of adhesions in the uterine cavity with scissors or biopsy forceps under hysteroscopic guidance has the advantage of removing less endometrium and avoiding possible complications related to energy sources [41]. According to a systematic review and meta-analysis (2017) by Yan Y. et al., intrauterine spherical stents with carboxymethyl cellulose and balloons with amniotic graft lyophilisate are the most effective anti-relapse therapy after surgical adhesiolysis [67].

For patients with AS and “thin” endometrium, there are practically no effective pharmacological preparations with high regenerative and anti-inflammatory potential today [68]. Applied approaches (hormone therapy, sildenafil citrate, aspirin, pentoxifyl line, tocopherol, tamoxifen, growth factors, etc.) often fail to solve the core problem—restoration of regenerative properties of the endometrium and its functional activity. One of the promising approaches to stimulate the processes of regeneration and angiogenesis in traumatic or ischemic tissue damage, including the endometrium, is cell therapy with MMSCs [69].

## 4. Effect of Multipotent Mesenchymal Stromal Cells on Endometrial Regeneration

MMSCs are fibroblast-like self-renewing cells belonging to peri- and postnatal stem cells, which can be isolated from various tissue sources (bone marrow, muscle tissue, liver, placenta, umbilical cord, adipose tissue, pancreas, cornea, retina, endometrium, intestines, and peripheral blood) and can differentiate into strictly defined types of specialized cells of a given tissue [70,71]. MMSCs are a private source of extracellular vesicles (EVs) used and studied in experiment and in clinical practice due to their prevalence and relative ease of cultivation [72]. Compared to other human immortalized lines, MMSCs have undeniable advantages: they represent a normal cell type in the primary culture, actively proliferate, and have not undergone oncogenic transformation [73]. Isolation of MMSCs results in high cell yields, is technically simple, inexpensive, and requires solving fewer ethical issues. MMSCs of various origins are becoming increasingly popular as a platform for testing the cytotoxicity and genotoxicity of pharmaceutical preparations as an alternative to lymphocytes and immortalized cell lines, and are also actively used as a convenient source of EVs collected from their culture media [73].

Experimental studies demonstrated the ability of MMSCs to influence endometrial regeneration. The introduction of MMSCs adipose tissue into the uterus and intraperitoneally in rat models with endometrium damaged by trichloroacetic acid led to an increase in vascularization and cell proliferation in the damaged areas. At the same time, with systemic administration of labeled MMSCs, in fact, they were not recorded in the endometrium, in contrast to their direct transplantation into the endometrium, where the proportion of labeled cells was 4–6% [74]. In studies on the effect of MMSCs from the bone marrow and umbilical cord on the formation of the uterine scar, their stimulating effect on vascular remodeling and the formation of de novo vessels in the rumen was shown [75,76]. The stimulating effect of MMSCs on angiogenesis and migration of placental endothelial cells was demonstrated through the activation of pro-angiogenic proteins (angiogenin, angiopoietin-1/2, GRO (growth-regulating oncogene), IL-6, IL-8, MCP-1, thrombopoietin, TIE-2 (angiopoietin receptor), TIMP-1/2 (tissue matrix metalloproteinase inhibitor), and VEGFB). In an in vitro study by Zhu H. et al., MMSCs isolated from menstrual blood contributed to increased proliferation of endometrial stromal cells, suppressing myofibroblast differentiation by inducing the Hippo/TAZ signaling pathway and suppressing TGF-β [77]. Transplantation of bone marrow MMSCs into the uterine cavity of an experimental model of “thin” rat endometrium, while increasing the thickness of the endometrium, also activated the expression of markers of regeneration (cytokeratin, vimentin) and receptivity (integrin αγβ3 and LIF), and also led to an increase in anti-inflammatory (IL-6, bFGF) and a decrease in pro-inflammatory (IL-1β, TNF-α) cytokines [78]. It is assumed that the effect of cell therapy in regeneration is to normalize homeostasis (growth factors, cytokines, etc.), stop the inflammatory process, as well as activate angiogenesis and resident stem cells.

In addition, the website of the US National Library of Medicine (NLM) at the National Institutes of Health (NIH) currently lists more than 1000 clinical studies involving MMSCs [79]. A number of publications confirmed the anti-inflammatory, anti-fibrotic, and immunomodulatory properties of cell therapy in women with damaged endometrium. Data on the successful restoration of the endometrium in a woman with refractory AS after repeated curettage using cell therapy with autologous MMSCs (1 × 10^7^) from the bone marrow through irrigation of the uterine cavity were published. Published data on the successful restoration of the endometrium in a woman with refractory SA after irrigation of the uterine cavity with autologous MMSCs (1 × 10^7^) from the bone marrow. Despite only a slight increase in the thickness of the endometrium (from 2.5 to 3.2 mm), pregnancy occurred spontaneously 4 months after cell therapy [80]. As part of a pilot trial on the use of MMSCs from menstrual blood for the treatment of AS, seven patients with refractory AS experienced uterine irrigation with autologous cells obtained on the second day of the menstrual cycle and cultured for a two-week period. It is important to note that in order to improve the penetration of MMSCs, small incisions were made on the endometrium before their introduction (endometrial scratching). Hormone therapy was carried out with estradiol valerate at a dose of 6 mg per day for 14 days after menstrual blood sampling and 4 mg per day for 21 days after the introduction of MMSCs. Analysis of ET showed a positive trend: in five patients, TE increased from 3–4 mm to 7–8 mm (*p* ˂ 0.001). In the case of slow growth of the endometrium, the introduction of MMSCs was repeated; two patients ended up with conception and normal pregnancies [81].

One of the potential factors affecting the effectiveness of MMSCs cell therapy in tissue regeneration is the low long-term engraftment of transplanted MMSCs in target tissues [82]. Some microenvironmental factors, hypoxia, washing out of cells from the injection site, and fibrosis can impair the viability of transplanted stem cells in damaged tissue [83]. A potential solution for increasing the viability of stem cells can be their transplantation using biomaterials, such as collagen scaffolds, hyaluronic acid gels, hydrogels, etc., to regenerate damaged tissues. The use of such biomaterials is aimed at controlled delivery of MMSCs, maintaining their viability, ensuring their slow release, creating conditions for successful migration, proliferation and differentiation of endometrial cells, as well as reaching an anti-adhesive effect. The combined effect of biomaterials and MMSCs cell therapy was demonstrated not only in a number of experimental studies [82], but is also now clinically confirmed. The introduction of umbilical cord MMSCs in biodegradable collagen scaffolds into the uterine cavity of 26 patients with recurrent intrauterine synechia, showed an increase in the ET in 100% of cases (from 4.46 ± 0.85 to 5.74 ± 1.2 mm (*p* < 0.01), led to the onset of pregnancy in 10 women (38%), of which eight pregnancies ended in the birth of healthy children [84]. Transplantation of a collagen scaffold with cord blood MMSCs to 15 patients with “thin” endometrium and infertility during two menstrual cycles resulted in an increase in ET from 4.08 ± 0.26 mm to 5.87 ± 0.77 mm in all patients (*p* < 0.001), pregnancy in 20% (2 spontaneous, 1—in the IVF program), live birth—in 13% of patients [85]. However, despite studies of the therapeutic potential of MMSCs, the complex contribution of MMSCs intercellular communication to regeneration processes remains insufficiently studied. Previously, it was believed that under the action of chemoattractants (CXCL12, CXCL8, CCL10, etc.) expressed in the damaged area, a part of MMSCs is mobilized and the dead cells are, subsequently, replaced by incorporation and differentiation into functional endometrial cells [86]. At the same time, the detection of an extremely low percentage of administered MMSCs in the endometrium [87] indicates the validity of the paracrine hypothesis, explaining the therapeutic effect after cell therapy. It is based on paracrine stimulation, that is, the ability of MMSCs to stimulate microenvironment cells through direct intercellular contact, secretion of biologically active molecules into the extracellular space, primarily EVs, as well as growth factors, chemokines, cytokines, etc. [88]. This is confirmed by the available data on the regenerative potential of MMSC EVs in case of damage to the kidneys, heart, liver, uterus, and nervous tissue [85,89,90,91,92,93]. Examples of MMSC introductions are summarized in Table 1.

## 5. Effect of MMSC-Derived Extracellular Vesicles on Endometrial Regeneration

EVs are extracellular bodies up to 4000 nm in size, which are formed during the normal or pathological process of vital activity by any body cells after activation of intracellular signaling cascades and at the initial stages of apoptosis. Today, EVs are considered as a key link in the new paradigm of intercellular communication, which consists of three main parts: the proximal element (cell source of signaling molecules), the transport element, which moves to the target to obtain a biological response, and the distal element (cell receiving and processing information). EVs regulate the activity of both proximal and distal target cells, including translational activity, angiogenesis, proliferation, metabolism, and apoptosis [94]. Recent studies showed that the amount and composition of EVs in the blood in a particular pathology is very specific. For example, a unique EVs profile is characteristic of various types of cancer [95], neurodegenerative diseases [96], and prion diseases [97]. Changes in the composition and content of EVs in blood plasma were also demonstrated for normal pregnancy [98] and gestational diabetes mellitus [99].

The function of EVs is to provide intercellular interactions and transport of various active molecules. EVs, the source of which are MMSCs, are able to participate in stem cell differentiation, innate and acquired immunity, tissue repair, and angiogenesis [4]. EVs are subdivided into exosomes (about 40–200 nm in diameter), microvesicles (100–2000 nm), and apoptotic bodies (200–4000 nm) [100].

Exosome membranes are characterized by specific markers (CD63, CD9 CD81), inside there are cytoplasmic inclusions, such as microRNA, peptide hormones, cytoplasmic and organelle-specific proteins. Unlike microvesicles, which are formed by protrusion of a portion of the plasma membrane, exosome biogenesis occurs in multivesicular bodies, or late endosomes [101].

Apoptotic bodies are released by dying cells; they are also capable of transporting biologically significant molecules, but these functions are not well understood [102]. Some researchers also isolated exomers with a diameter of about 35 nm [103], which is close to the theoretical minimum size of bodies with a phospholipid membrane (10–20 nm). Exomers differ from EVs in protein and lipid composition [104]. EVs contain mRNA, non-coding RNA, DNA, as well as cytoskeletal proteins, signaling proteins and lipids, organelles or their parts protected by a membrane from degradation until the content is “delivered” to the target cell, to which they bind with high specificity [4,105]. In this case, the lipid bilayer of the EVs membrane is able to bind to one or another cell, depending on which cell became the source of EVs [106]. After that, EVs induce a cascade of signal transduction processes in the cell. EVs are heterogeneous in nature, while the same biochemical and biophysical effects may be inherent in their various subtypes. That is why it is important to be careful when developing methods for isolating EVs and describing them [107].

It is known that EVs are involved in the processes of human reproduction at the levels of implantation and embryonic development [12,108]. Successful pregnancy requires continuous interaction at the molecular level, including endo-, para-, and autocrine factors. EVs provide a possible direct and dynamic pathway for communication between the embryo and the maternal organism at the stages of early embryogenesis, embryonic modulation of endometrial receptivity, and trophoblast invasion [109].

According to some reports, the secretion of EVs in the endometrium of women with infertility may be impaired. Analysis of the proteome of fluid from the uterine cavity in patients with habitual implantation failures revealed significant changes in the expression of several proteins, some of which are associated with EVs [110]. Exosome-associated proteins CD63 and CD9 were proposed as possible biomarkers of impaired endometrial receptivity in unexplained infertility [111].

The paracrine mechanisms in the endometrium mediated by EVs may be involved in ensuring successful implantation [112]. MMSC-derived EVs, as well as stromal and epithelial cells of the endometrium, contain numerous proteins involved in the processes of embryonic development and implantation [113]. There is evidence that exosomes of the uterine cavity fluid promote cell proliferation in the implantation zone, regulating gene expression due to microRNAs such as miR-200c and miR-30d, and interferon-τ [114,115,116].

Today, an exosomal mechanism for improving endometrial receptivity, in which EVs serve as a means for delivering chorionic gonadotropin to epithelial cells, was also proposed [117]. However, this idea was not yet clinically confirmed.

The clinical use of EVs in comparison with MMSCs themselves in order to restore the morphological state of the damaged endometrium and its receptivity provides a number of advantages, including a better safety profile, reduced immunogenicity, and the ability to cross biological barriers [11]. There is evidence that EVs are involved in tissue regeneration not only through the activation of progenitor/stem cells and angiogenesis, but also through MET stimulation, contributing to the suppression of proliferation and migration of fibroblasts and endothelial cells through various signaling pathways [118]. At the same time, it should be noted that EVs of other genesis can be involved in pathological EMT [119]. In particular, EVs isolated from adenomyosis tissue induce EMT processes, which was confirmed by a decrease in the expression of E-cadherin and cytokeratin 19 and an increase in the expression of vimentin and ZEB1, and increase the invasiveness of epitheliocytes, which may contribute to the progression of this disease [120]. MMSCs are involved in the regulation of the inflammatory process, initiating the transition of inflammation to repair. The mechanism of the anti-inflammatory effect of EVs is little studied and is important for the restoration of the endometrium in cases of endometrium regeneration impairment, which developed under conditions of endometritis. Xin L. et al. showed that intrauterine transplantation of exosomes isolated from umbilical cord MMSCs on a collagen scaffold to experimental rats with damaged endometrium contributed to its regeneration, collagen remodeling, increased expression of steroid hormone receptors (ERα and PR), inhibition of inflammation, and restoration of fertility. According to researchers, this positive result was primarily due to the immunomodulatory effect of miRNA exosomes due to the polarization of CD163+ M2 macrophages, reducing inflammatory manifestations, and enhancing the anti-inflammatory response in vivo and in vitro [121]. This work did not deal with the evaluation of MET markers and signaling pathways through which EVs exert their antifibrotic effect. The effects of the introduction of EV are presented in Table 2.

In a study of AS on an experimental rat model, adipose tissue MMSCs exosomes maintained the normal structure of the organ, promoted endometrial regeneration and collagen remodeling, and increased the expression of integrin β3, LIF, and VEGF. The positive effect was confirmed by an increase in endometrial receptivity [122].

A promising therapeutic approach may be the use of MMSCs exosomes from the bone marrow, which are capable of transporting miR-340 [123] and miR-29a [124] microRNAs [124] into endometrial cells, thereby realizing an antifibrotic effect. An increase in the expression of matrix metalloproteinases (MMP-2, MMP-9) and suppression of the expression of their tissue inhibitor (TIMP-2) with the introduction of exosomes isolated from endometrial MMSCs were also described. This made it possible to more rapidly enhance the processes of proliferation and vascularization and reduce the severity of fibrosis in an animal model (rats) of AS, compared with the use of MMSCs themselves [125].

Exosomes of bone marrow stem cells, such as these cells themselves, contribute to the restoration of the endometrium after damage—in a study on experimental animals (rabbits), activation of the TGF-β1/Smad signaling pathway by exosomes contributed to the reversal of EMT [126]. It was noted that a possible modification of MMSCs—exosome donors with hyperexpression of cytokine cardiotrophin-1—can more effectively contribute to the restoration of the endo- and myometrium in a situation where neovascularization can increase endometrium receptivity [127].

MMSCs can also become a source of apoptotic bodies, which, along with exosomes, can promote endometrial regeneration and restore fertility. This class of EVs is also capable of inducing macrophage immunomodulation, cell proliferation, and angiogenesis [128].

Due to the fact that conclusions regarding the assessment of the effect of various types of EVs on endometrial regeneration were obtained from a limited number of experimental studies using a variety of animal models, it is currently difficult to draw a conclusion about their effectiveness in humans. In addition, the uniqueness of the endometrium and its hormonal control in each species raises the question of the possibility of extrapolating the obtained data to the regeneration of the human endometrium. However, due to the ethical and technical issues associated with conducting such clinical trials, continued evaluation of the efficacy and safety of EVs therapy in vitro and in vivo may warrant the initiation of clinical trials.

## 6. Conclusions

**Figure 2:**
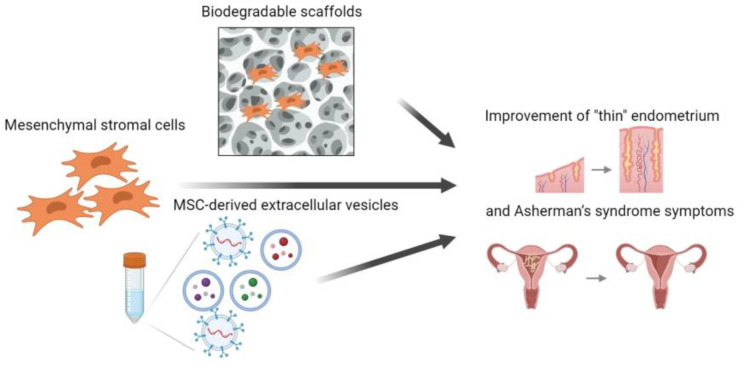
Schematic illustration of the application of multipotent mesenchymal stromal cells, their extracellular vesicles and their combinations with bioengineering techniques (biodegradable scaffolds) in therapy of “thin” endometrium and Asherman’s syndrome.

Thus, EVs can be considered not only as biologically active substances that regulate the functional potential of the endometrium and participate in the processes associated with embryo implantation, but also from the standpoint of an alternative to the cellular method of therapy. Further study of endometrial EVs and uterine cavity fluid will allow better characterization of implantation processes and identification of new biomarkers, which will make it possible to choose the best moment for embryo transfer into the uterine cavity. The assessment of the possibility of using MMSC-derived EVs for intercellular communication in biomedicine and the development of cell-free therapy in combination with bioengineering technologies will become a promising direction in solving the problem of infertility associated with a decrease in the receptivity and regenerative potential of the endometrium (Figure 2).

## Figures and Tables

**Table 1:** Effects of multipotent mesenchymal stromal cells at “thin” endometrium and Asherman’s syndrome.

Authors	Type of MMSC	Model	Results	References
Kilic, S. et al.	MMSCs from the bone marrow and umbilical cord	Rat model of AS	Increased endometrial vascularization and decreased fibrosis	[74]
Pekarev, O.G. et al.	Human umbilical cord MSC	Rat model of a uterine scar	Stimulating effect on vascular remodeling and the formation of de novo formed vessels in the uterine scar	[75]
Zhu, H. et al.	Menstrual stem cells	In vitro	Increased proliferation of endometrial stromal cells, suppression of myofibroblast differentiation	[77]
Zhao, J. et al.	Autologous bone marrow derived MSC	Rat model of “thin” endometrium	Increased the ET, activated the expression of markers of regeneration and receptivity, anti-inflammatory effects	[78]
Tan, J. et al.	Autologous menstrual blood-derived stromal cells	Patient’s with severe AS	Increased the ET (71%), pregnancy (71%), live birth (29%)	[81]
Zhao, Y. et al.	Autologous bone marrow derived MSC	Patient’s with intrauterine adhesions	Restoration of the endometrium in a woman with refractory AS	[80]
Cao, Y. et al.	Umbilical cord MSCs on collagen scaffolds	Patients with Recurrent Uterine Adhesions	Increased in the ET (100%), pregnancy (38%)	[84]
Zhang, Y. et al.	Umbilical cord MSC on collagen scaffolds	Patients with AS	Increase in ET (100%), pregnancy (31%), live birth (12%)	[85]

**Table 2:** Effects of MMSCs-derived extracellular vesicles at “thin” endometrium and Asherman’s syndrome.

Authors	Type of Extracellular Vesicles	Model	Results	References
Xin, L. et al.	Umbilical cord MMSCs-derived exosomes on a collagen scaffold	Injured rat uterus In vitro	Induction of endometrial regeneration, collagen remodeling, increased ER α/RP expression and restoration of fertility.	[121]
Zhao, S. et al.	Exosomes Derived from Adipose MSCs	Rat Model of Intrauterine Adhesions	Maintenance of normal uterine structure, activation of endometrial regeneration and collagen remodeling, increased expression of integrin-β3, LIF and VEGF, increased endometrial receptivity and fertility	[122]
Xiao, B. et al.	Bone marrow MMSCs-derived exosomes	Injured rat uterus In vitro	Antifibrotic effect (improved functional recovery and suppression of collagen 1α1, α-SMA and TGF-β1,suppression of increased expression of fibrotic genes induced by TGF-β1)	[123]
Tan, Q. et al.	Bone marrow MSC-derived exosomes	Mouse model of intrauterine adhesions In vitro	Activation of cell proliferation and cell migration in vitro, repair of damaged endometrium in a mouse model	[124]
Saribas, G.S. et al.	Exosomes from uterus derived MSC	Rat model of AS	Increase in proliferation and vascularization, decrease in fibrosis in the uterus	[125]
Yao, Y. et al.	MMSC-derived exosomes	Rabbit model of intrauterine adhesions	Repair of damaged endometrium by reversing EMT via the TGF-β1/Smad signaling pathway	[126]
Zhu, Q. et al.	Exosomes derived from CTF1-modified bone marrow stem cells	Injured rat uterus	Activation of tissue regeneration of the endometrium and myometrium, improvement of endometrial receptivity, stimulating neovascularization	[127]

## Data Availability

Data available in a publicly accessible repository.

## Abbreviations

AAGL	American Association of Gynecologic Laparoscopists
ART	assisted reproductive technique
AS	Asherman’s syndrome
AXIN2	axis inhibition protein 2
BMDSC	bone marrow-derived stem cell
EGF	epithelial growth factor
ERK	extracellular-signal-regulated kinase
ERα	estrogen receptor α
ET	endometrial thickness
EV	extracellular vesicle
FGF2	fibroblast growth factor 2
GRO	growth-regulating oncogene
IL	interleukin
MARK	mitogen-activated protein kinase
MET	mesenchymalmal-epithelial transition
MMP	matrix metalloproteinase
MMSC	multipotent mesenchymal stromal cell
NF-κB	nuclear factor-κB
NLM	National Library of Medicine
PR	progesterone receptor
ROS	reactive oxygen species
SHG	sonohysterography
Snai 1,2,3	Snail family transcriptional repressor 1,2,3
SSEA-1	stage-specific embryonic anti-gene-1
TAZ	transcriptional coactivator with PDZ-binding motif
TGF-1	transforming growth factor 1
TGFα	transforming growth factor α
TIE-2	angiopoietin receptor
TIMP-1/2	tissue matrix metalloproteinase inhibitor
VEGFB	vascular endothelial growth factor
WT1	Wilms’ tumour 1
YAP	Yes-associated protein
NIH	National Institutes of Health
